# Self-Expandable Metal Stent Placement for Closure of a Leak after Total Gastrectomy for Gastric Cancer: Report on Three Cases and Review of the Literature

**DOI:** 10.1155/2014/409283

**Published:** 2014-10-09

**Authors:** Dario Raimondo, Emanuele Sinagra, Tiziana Facella, Francesca Rossi, Marco Messina, Massimiliano Spada, Guido Martorana, Pier Enrico Marchesa, Rosario Squatrito, Giovanni Tomasello, Attilio Ignazio Lo Monte, Giancarlo Pompei, Ennio La Rocca

**Affiliations:** ^1^Gastroenterology and Endoscopy Unit, Fondazione Istituto San Raffaele-G. Giglio, Contrada Pietra Pollastra Pisciotto, 90015 Cefalù, Italy; ^2^Ph.D. Course in Surgical Biotechnology and Regenerative Medicine, University of Palermo, Piazza delle Cliniche 2, 90100 Palermo, Italy; ^3^Euro-Mediterranean Institute of Science and Technology (IEMEST), Via Emerico Amari 123, 90100 Palermo, Italy; ^4^Surgery Unit, Fondazione Istituto San Raffaele-G. Giglio, Contrada Pietra Pollastra Pisciotto, 90015 Cefalù, Italy; ^5^Oncology Unit, Fondazione Istituto San Raffaele-G. Giglio, Contrada Pietra Pollastra Pisciotto, 90015 Cefalù, Italy; ^6^Internal Medicine Unit, Fondazione Istituto San Raffaele-G. Giglio, Contrada Pietra Pollastra Pisciotto, 90015 Cefalù, Italy; ^7^Department of Surgical and Oncological Disciplines, University of Palermo, Piazza delle Cliniche 2, 90100 Palermo, Italy; ^8^Pathology Unit, Fondazione Istituto San Raffaele-G. Giglio, Contrada Pietra Pollastra Pisciotto, 90015 Cefalù, Italy; ^9^Unità Operativa di Medicina Interna e Trapiantologia, Ospedale San Raffaele, Via Olgettina 60, Milano, Italy

## Abstract

In the setting of the curative oncological surgery, the gastric surgery is exposed to complicated upper gastrointestinal leaks, and consequently the management of this problem has become more critically focused than was previously possible. We report here three cases of placement of a partially silicone-coated SEMS (Evolution Controlled Release Esophageal Stent System, Cook Medical, Winston-Salem, NC, USA) in patients who underwent total gastrectomy with Roux-en-Y end-to-side esophagojejunostomy for a gastric adenocarcinoma. The promising results of our report, despite the small number of patients, suggest that early stenting (through a partially silicone-coated SEMS) is a feasible alternative to surgical treatment in this subset of patients. In fact, in the treatment of leakage after total gastrectomy, plastic stents and totally covered metallic stents may not adhere sufficiently to the esophagojejunal walls and, as a result, migrate beyond the anastomosis. However, prospective studies with a larger number of patients might assess the real effectiveness and safety of this procedure.

## 1. Introduction

In the setting of the curative oncological surgery, the gastric surgery is exposed to complicated upper gastrointestinal leaks, and consequently the management of this problem has become more critically focused than was previously possible. The management of a patient with an upper gastrointestinal leak is complex and often involves treatment of sepsis, organ failure, and nutritional deficits in addition to treating the underlying leak [1]. The use of stents in the management of postoperative leaks has been reported with variable success [2–10].

We report here three cases of placement of a partially silicone-coated SEMS (Evolution Controlled Release Esophageal Stent System, Cook Medical, Winston-Salem, NC, USA) in patients who underwent total gastrectomy with Roux-en-Y end-to-side esophagojejunostomy for a gastric adenocarcinoma.

## 2. Case Report 1

**Figure 1:**
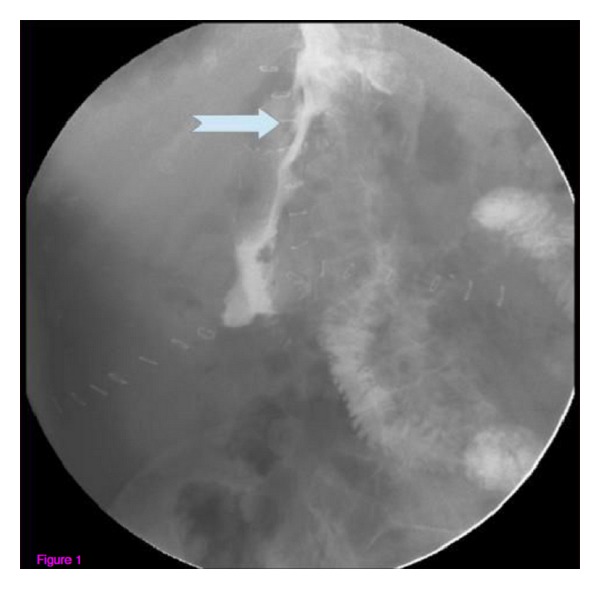
Gastrografin esophagography showing a 2 cm fistula at the anastomosis.

**Figure 2:**
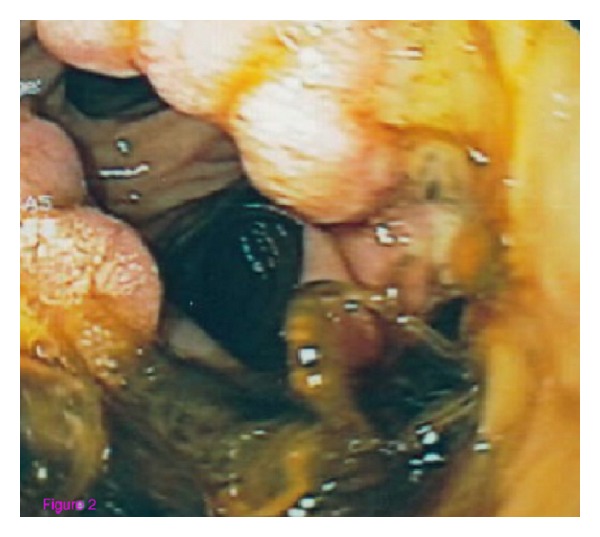
Endoscopic view of the fistula.

**Figure 3:**
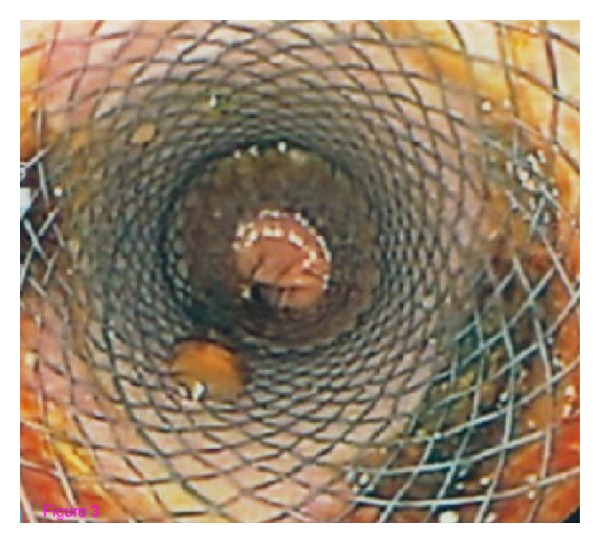
Endoscopic view of the placement of the stent.

**Figure 4:**
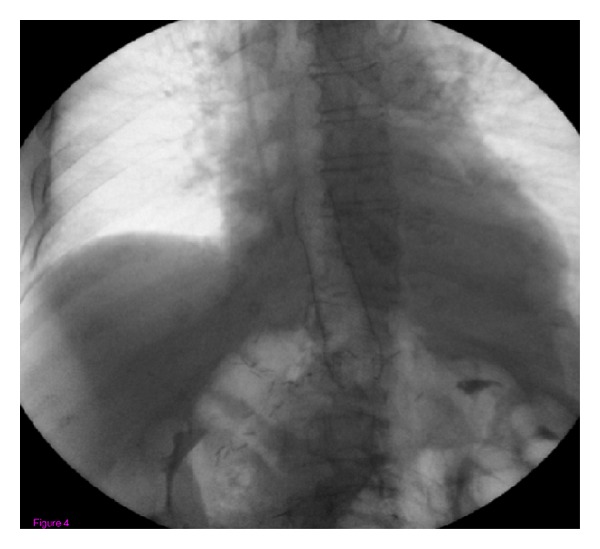
Gastrografin esophagography showing the absence of contrast extravasation, after the placement of the stent.

A 52-year-old woman was admitted to our hospital for abdominal pain and weight loss. Esophagogastroduodenoscopy (EGDS) showed a gastric lesion, which on histology was revealed to be a gastric adenocarcinoma. As a result, the patient underwent total gastrectomy with Roux-en-Y end-to-side esophagojejunostomy. A stapling esophagojejunostomy was performed: the anvil was secured in the esophagus with a purse-string suture; subsequently an end anastomosis stapler was inserted through the distal end of the Roux limb. The anastomosis was made on the antimesenteric side of the bowel. Once the anastomosis was complete, the end of the Roux limb was amputated with a single firing of a gastrointestinal anastomosis stapler. The enteric staple line was reinforced with interrupted Lembert sutures of 3–0 silk. Five days later, due to the onset of nausea and abdominal pain, EGDS was performed, showing a 2 cm fistula at the anastomosis, about 38 cm from the mouth (Figure 2). This was confirmed by gastrografin esophagography. The patient was started on total parenteral nutrition (TPN) (Figure 1) and empirical antibiotic therapy. The abdominal cavity drainage was performed maintaining a drainage tube for 15 days, which was removed; after that the disappearance of the abdominal effusion was demonstrated through abdominal ultrasound. Having deemed clipping treatment for this fistula unfeasible, we decided to insert a partially silicone-coated SEMS (Evolution Controlled Release Esophageal Stent System). The Evolution stent (Cook, Bloomington, IN, USA) is available as a partially or fully covered SEMS. The stent is encased (Figure 3) with silicone on its exterior and interior surfaces to prevent tumor ingrowth. A unique feature of Evolution delivery system is that it enables a controlled release and recapturability feature with a “point of no return” indicator. With each squeeze of the stent system's trigger-based introducer, a proportional length of the stent is deployed or recaptured. The diameter of the Evolution stent employed was 25 mm and 20 mm at the flare and at the shaft, respectively; the length was 10 cm. We chose this type of partially covered metallic stent in order to avoid the kind of stent migration described in some reports [9, 11, 12]. The stent was removed after six weeks. EGDS showed closure of the fistula. No clinical complications were observed, and the patient was able to start normal per os nutrition 7 days after the stent placement, once an upper X-ray series with gastrografin secured the disappearance of the contrast extravasation (Figure 4).

## 3. Case Report 2

Like the previous patient, a 71-year-old woman was admitted to our hospital for abdominal pain and weight loss. EGDS showed a gastric lesion, which on histology was revealed to be a gastric adenocarcinoma, and the patient underwent total gastrectomy with Roux-en-Y end-to-side esophagojejunostomy. A stapling esophagojejunostomy was performed, according to the aforementioned procedure. Eight days later, due to the onset of fever and abdominal pain, EGDS was performed, showing a 1.7 cm fistula at the anastomosis, about 40 cm from the mouth. This was confirmed by gastrografin esophagography. The patient was started on TPN and empirical antibiotic therapy. The abdominal cavity drainage was performed maintaining a drainage tube for 15 days, which was removed; after that the disappearance of the abdominal effusion was demonstrated through abdominal ultrasound. Having deemed clipping treatment for this fistula unfeasible, we decided, also in this patient, to insert a partially silicone-coated SEMS (Evolution Controlled Release Esophageal Stent System). The patient was able to start normal per os nutrition 7 days after the stent placement, once an upper X-ray series with gastrografin secured the disappearance of the contrast extravasation. The stent was removed after six weeks. EGDS showed closure of the fistula. Unfortunately, the patient died of a pulmonary embolism, ten days after stent removal.

## 4. Case Report 3

**Figure 5:**
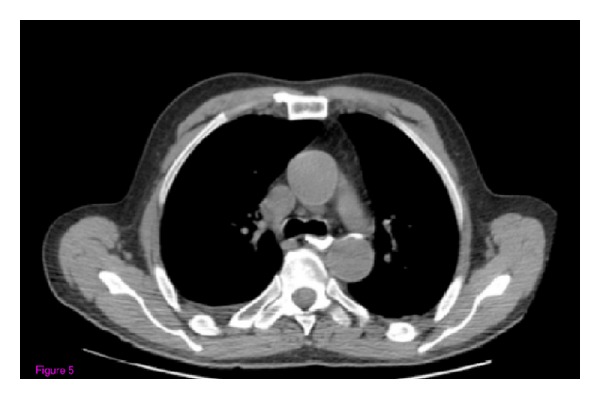
CT scan with gastrographin showing the contrast extravasation.

Finally, a 66-year-old man was admitted to our hospital with severe anemia (6 g/dL), abdominal pain, and weight loss. Like in the cases reported above, EGDS showed a gastric lesion, which on histology was revealed to be a gastric adenocarcinoma, and the patient underwent total gastrectomy with Roux-en-Y end-to-side esophagojejunostomy. A stapling esophagojejunostomy was performed, according to the aforementioned procedure. Seven days later, EGDS was performed, due to the onset of severe nausea and fever, showing a 1.8 cm fistula at the anastomosis, about 38 cm from the mouth. This was confirmed by CT scan with gastrografin. The patient was started on TPN and empirical antibiotic (Figure 5) therapy. The abdominal cavity drainage was performed maintaining a drainage tube for 15 days, which was removed; after that the disappearance of the abdominal effusion was demonstrated through abdominal ultrasound. Having deemed clipping treatment for this fistula unfeasible, we decided, also in this patient, to insert a partially silicone-coated SEMS (Evolution Controlled Release Esophageal Stent System). The stent was removed after six weeks. Gastrografin esophagography showed no further contrast extravasation, and EGD showed closure of the fistula. No clinical complications were observed, and the patient was able to start normal per os nutrition 7 days after the stent placement, once an upper X-ray series with gastrografin secured the disappearance of the contrast extravasation.

## 5. Discussion

The treatment of symptomatic leaks in patients who have undergone esophagojejunostomy is challenging, and leakage from the jejunal stump can be a potentially serious complication. Therapeutic options are surgical repair or resection or conservative management with cessation of oral intake and antibiotic therapy. Open surgical reintervention is associated with considerable risk, particularly in depleted patients [13].

This report provides further evidence that stenting for upper gastrointestinal leaks is a simple and robust technique and has the definite advantage of allowing early recommencement of enteral nutrition. In our small series, we report only three cases of anastomotic leaks after total gastrectomy for gastric cancer, which occurred in our center from January 2010 to December 2013, which were treated with SEMS placement, with a technical and clinical success rate of 100%. Stenting should be considered early in the management of patients with evolving sepsis from gastrointestinal leakage even where the defect is large. It is suitable for combination with laparoscopic, thoracoscopic, or open drainage when contamination is significant. Previous reports have suggested the effectiveness and the safety of this procedure [1, 14]. In the study performed by Babor and coworkers, in the setting of bariatric surgery, seven patients with staple line and anastomotic dehiscences and a single case of Boerhaave syndrome were treated by using a removable, polyester covered self-expanding metal stent (ELLA Boubella, Ella-CS, Hradec, Czech Republic). All patients had active severe sepsis and significant contamination in the abdomen or thorax at the time of stenting. In 4 cases, the stent was sutured in place with dissolvable synthetic sutures with suture bites incorporating the full thickness of the gut wall and the stent itself to prevent stent migration. All patients showed resolution of their intra-abdominal sepsis and were able to resume an oral diet after stenting; all stents were retrieved endoscopically after clinical resolution of the leak. Stent migration after leak resolution was observed in 3 patients. In patients with large defects or minimal anatomic barriers to stent migration, suture fixation stabilized the stent; there were no episodes of persistent leak or development of stricture in this series [1]. Eisendrath and coworkers also evaluated, in the setting of bariatric surgery, in a series of twenty-one patients which underwent endoscopic treatment for persisting large anastomotic leaks before considering redo surgery, whether endoscopic treatment may reduce reoperation rates [15]. In these patients, partially covered SEMS were used, followed by additional endoscopic procedures if the SEMS failed. SEMS insertion led to 62% (13/21) primary closures. Complementary endoscopic treatment led to 4 secondary closures. Total success rate was 81% (17/21). Interestingly, the success rates of endotherapy were 100% (8/8) in the gastric bypass group, 62.5% (5/8) in the SDS group, 75% (3/4) in the sleeve gastrectomy group, and 100% (1/1) for the Scopinaro procedure; furthermore, gastrocutaneous fistulas on a sleeve suture seemed to be the most difficult condition to treat (successfully treated in 60% of cases (6/10)) [15].

Recently, Shim and coworkers performed a prospective study to assess the clinical characteristics and therapeutic outcomes of SEMS and nonstent endoscopic therapy (NSET, which includes fibrin glue injection, endoclip, and other devices) for treatment of anastomotic leaks after total gastrectomy with the aim of assisting endoscopists in choosing a treatment method [16]. 13 patients treated with SEMS (with a total of 16 SEMS placement sessions) and 14 patients treated with NSET (with a total 21 NSET sessions) for anastomotic leaks after total gastrectomy were enrolled onto the study. At the end of the study, the successful sealing rate at the first attempt by SEMS was significantly better than that of NSET (80.0 versus 28.6%, *P* = 0.036), whereas the successful sealing rate after multiple endoscopic treatments was not statistically different (80.0 versus 64.3%, *P* = 0.653). The main reason for reintervention with SEMS was complications and with NSET was nonseal (*P* = 0.004). Interestingly, clinical outcomes including length of hospital stay, endoscopic treatment-related mortality, and all-cause mortality were not significantly different between the 2 groups [16].

An area of uncertainty regards the real knowledge of the migration rate of partially covered SEMS in this subset of patients, as well as how long the stent should be left in place to allow removability and avoid complications.

In our series the complete closures of the anastomotic leaks were achieved after 6 weeks of stent indwelling using partially covered metal stents in all 3 patients.

In an article of systematic review on temporary stent placement for benign rupture or anastomotic leaks of the esophagus, twenty-five studies made up of 267 patients who were treated with endoscopic placement of fully covered or partially covered SEMS or plastic stents were analyzed; the mean indwelling time of partially covered SEMS was 6 weeks. It was concluded that covered stent placement for a period of 6–8 weeks was safe and effective for benign esophageal ruptures and anastomotic leaks to heal [17]. An article from Lee and coworkers reported that healing of leaks after endoscopic placement of covered stents in patients with anastomotic leaks occurred in 4 out of 6 patients after 2 weeks of stent indwelling [18], while an article from Schubert and coworkers reported that a complete closure of the leakage after silicone-covered, self-expanding polyester stents in patients with thoracic esophageal anastomotic leaks was achieved in 11 of 12 patients after stent removal with a median stent retrieval time of 4 weeks (range 2–8 weeks) [9]. On the other hand, Eubanks and coworkers reported that healing of leaks after endoscopic placement of covered stents in patients with anastomotic leaks after bariatric surgery was achieved at a mean of 33 days [19], while Edwards and coworkers reported that acute anastomotic leaks after Roux-en-Y bypass had completely healed at a median of 44 days when self-expanding polyester stents were used [20]. Finally, Fischer and coworkers recently reported that the median stent indwelling time after using a specially designed partially covered self-expandable metal stent in 11 patients with upper GI anastomotic leaks was 24 (range 18–41) days [21].

Although stent migration remains a common problem when using fully covered metal stents in patients with postoperative anastomotic leaks, several novel fixation procedures were reported to eliminate migration of the fully covered metal stents, such as a stent fixation technique with a bridle which was created by delivering umbilical tape through each nostril [22] or through a novel stent insertion technique with a newly designed proximal-releasing, self-expanding metallic stent (PR-SEMS) and transnasal endoscope that can enable stent insertion without fluoroscopy as a new method to prevent stent migration [18].

It is worth mentioning, in the fields of leaks and perforation of the upper gastrointestinal tract, the use of endoscopic placed vacuum sponge therapy. In the study performed by Schorsch and coworkers, 17 cases of anastomotic leakage and 7 cases of iatrogenic perforation due to interventional endoscopy or rigid panendoscopy with either intraluminal or intracavitary endoscopic vacuum therapy were treated [23]. In 23 of 24 cases, the endoscopic treatment was successful. The median duration of therapy was 11 days (range 4–46 days). All 7 cases of iatrogenic perforation and 16 of 17 anastomotic leakage cases were cured after a median therapy duration of 5 and 12 days, respectively. According to the authors' experience, it has seemed to be the best choice for iatrogenic perforations and has been a potent supplement in the management of anastomotic leakages [23].

It is remarkable that in all patients of our series the length of the fistula did not exceed 2 cm and that no further complications, like abscesses of sepsis, were present; as a consequence, the shortness of the fistulae and the lack of abscesses, together with the prompt antibiotic therapy, are probably further reasons for the rapid healing of the leaks.

The promising results of our report suggest that early stenting (through a partially silicone-coated SEMS) is a feasible alternative to surgical treatment in this subset of patients. In fact, in the treatment of leakage after total gastrectomy, plastic stents and totally covered metallic stents may not adhere sufficiently to the esophagojejunal walls and, as a result, migrate beyond the anastomosis. However, prospective studies with a larger number of patients might assess the real effectiveness and safety of this procedure.
